# Supplementary material for: Microstructural Dynamics of Polymer Melts during Stretching: Radial Size Distribution
Source: Polymers (Basel). 2023 Apr 26;15(9):2067. doi: 10.3390/polym15092067 (PMC10181331; doi:10.3390/polym15092067)
Supplement: Supplementary file 1 [file polymers-15-02067-s001.zip › polymers-2363171-supplementary.pdf]

Supporting Information

Microstructural dynamics of polymer melts during stretching: radial size distribution

Ming-Chang Hsieh[a], Yu-Hao Tsao[a], Yu-Jane Sheng[*,a] and Heng-Kwong Tsao[*,b]

Verification of Chain Uncrossibility

The dissipative particle dynamics (DPD) method is used in this study and the polymer chain is constructed by connected DPD beads. The original DPD parameters proposed by Groot and Warren [1] are too soft and provide no specific restrictions for steric interactions. Chain crossings may occur, resulting in partially phantom chains. The lack of chain entanglement greatly influences the dynamic properties of the polymer systems. Thus, the original DPD chains exhibit the Rouse dynamics [2,3] instead of the reputational dynamics as should be seen in polymer melts. When studying the microstructure dynamics of polymer melts during stretching and relaxation, entanglements between chains due to steric interactions should be explicitly present. Some methods were exploited to prevent the bond crossings in coarse-grained models [4-8]. Among them, Nikunen et al. [8] presented an easy and computationally efficient criterion to impose topological constraints leading to the uncrossability of polymeric chains for the DPD method. No additional forces are needed, and reputational dynamics can be reproduced, indicating the existence of proper chain entanglement behavior [8-11]. This concept has also been successfully employed to study the crystallization of semiflexible polymers in melts and solutions [12].

The criterion proposed by Nikunen et al. [8] is based on geometrical constraints. As shown in Figure S1, l is the bond length and l_{max} is the maximum value of l. r represents the distance between any two non-bonded beads (nonbonded length), and r_{min} denotes the minimum value of r. Although DPD beads are soft, they still possess an apparent excluded volume because of their repulsive potential. Since $r \geq r_{min}$, all beads effectively have an excluded volume with radius $r_{min}/2$. Therefore, if $\sqrt{2}r_{min} > l_{max}$ is fulfilled throughout the simulation, chain crossings will not take place. The condition can be satisfied by choosing the appropriate DPD parameters to maintain the uncrossability of polymeric chains, and at the same time, the large integration step can still be employed. Therefore, the repulsive interaction parameter is generally chosen to be large, such as

$a_{ij} = 150$, to increase r_{min} [12]. However, since the bead density within the system is kept the same as $\rho = 3$, it was found that a large value of the repulsive interaction parameter will not affect r_{min} significantly, but increase the value of l_{max} instead. In this study, we used the equilibrium bond length $r_{eq} = 0.4$ and stiff spring potential $k_s = 100$ to reduce l_{max}, while the repulsive interaction parameter is still set as $a_{ij} = 25$. The criterion of the uncrossability of polymeric chains is then proved to be satisfied as follows.

The bond length and the nonbonded length distributions of our simulation system can be acquired as P(l) and P(r). The comparison between l_{max} and $\sqrt{2}r_{min}$ is then performed by plotting the two normalized distributions P(l) and P($\sqrt{2}r$) together as shown in Figure S2. If the former is always on the left-hand side of the latter, the criterion of the uncrossability is inherently fulfilled. As one can see from Figure S2, the overlap between the two distributions is not evident at all and thus the criterion is essentially satisfied. Nontheless, as the region between the upper bound of P(l) and the lower bound of the P($\sqrt{2}r$) is greatly enlarged, the overlap is observed as shown in the inset of Figure S2. Fortunately, the total probability of $\sqrt{2}r < l_{max}$ is about 2×10^{-7}, which is negligibly small. Consequently, the uncrossability of polymer chains is effectively realized in our system. According to the previous study by Chang and Yethiraj, when chain-crossing incidents are rare, entanglements still persevere in polymer melts [13]. To verify our argument further, the evolution of two entangled polymer chains are simulated to examine the crossability. Videos S1 and S2 demonstrate that our choice of DPD parameters can indeed prevent chain-crossing events.

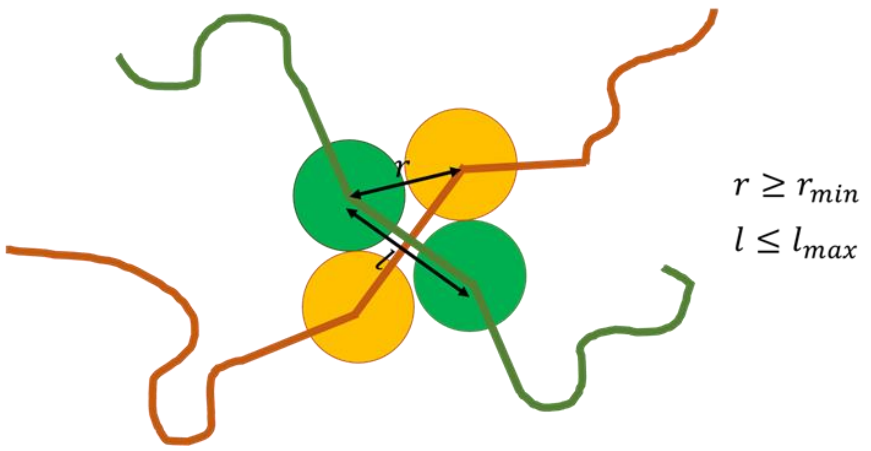

Figure S1. Schematics of two chains to check bond crossing conditions where r represents the distance between any two nonbonded beads, l denotes the bond length. r_{min} denotes the minimum value of r and l_{max} is the maximum value of l.

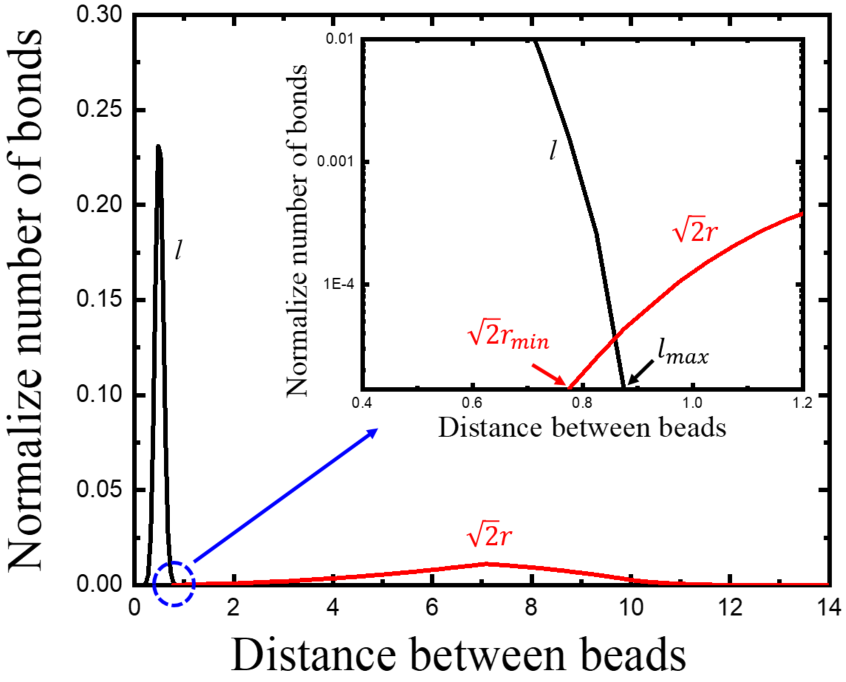

Figure S2. The bond length and the nonbonded length distribution of our simulation system.

[1] R. D. Groot and P. B. Warren, J. Chem. Phys., 1997, 107, 4423–4435.

[2] N. Spenley, Europhys. Lett., 2000, 49, 534.

[3] F. Lahmar and B. Rousseau, Polymer, 2007, 48, 3584–3592.

[4] A. Gooneie, S. Schuschnigg and C. Holzer, Polymers, 2017, 9, 16.

[5] Padding, J.T.; Briels, W.J. Uncrossability constraints in mesoscopic polymer melt simulations: Non-rouse behavior of C120H242. J. Chem. Phys. 2001, 115, 2846–2859.

[6] Pan, G.; Manke, C.W. Developments toward simulation of entangled polymer melts by dissipative particle dynamics (DPD). Int. J. Mod. Phys. B 2003, 17, 231–235.

[7] Sirk, T.W.; Slizoberg, Y.R.; Brennan, J.K.; Lisal, M.; Andzelm, J.W. An enhanced entangled polymer model for dissipative particle dynamics. J. Chem. Phys. 2012, 136, 134903.

[8] P. Nikunen, I. Vattulainen and M. Karttunen, Phys. Rev. E: Stat., Nonlinear, Soft Matter Phys., 2007, 75, 036713.

[9] A. Chertovich and P. Kos, J. Chem. Phys., 2014, 141, 134903.

[10] P. Espan˜ol and P. B. Warren, J. Chem. Phys., 2017, 146, 150901.

[11] A. Karatrantos, N. Clarke, R. J. Composto and K. I. Winey, Soft Matter, 2013, 9, 3877–3884.

[12] Kos, P. I.; Ivanov, V. A.; Chertovich, A. V. Crystallization of semiflexible polymers in melts and solutions. Soft Matter, 2021, 17, 2392.

[13] Chang, R.; Yethiraj, A. Can Polymer Chains Cross Each Other and Still Be Entangled? Macromolecules, 2019, 52, 2000–2006.

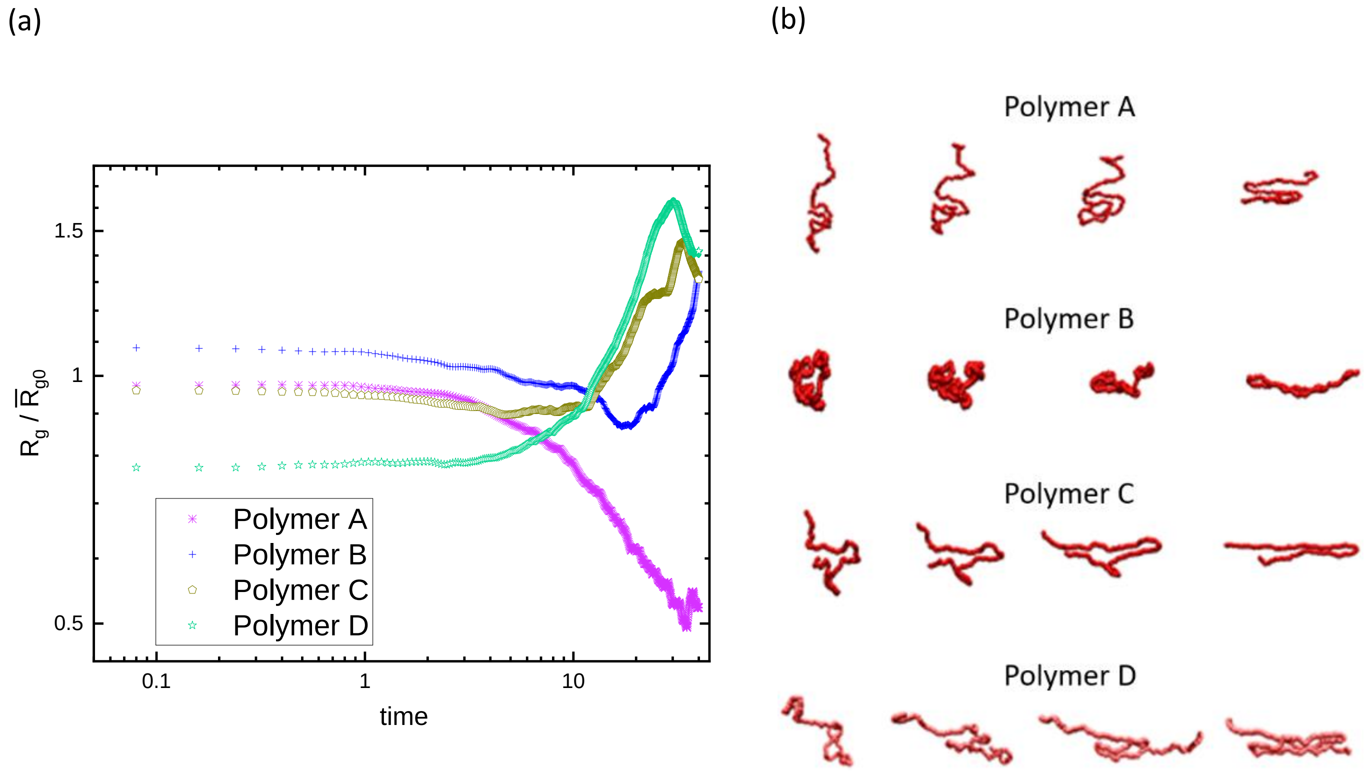

Figure S1. (a) The representative evolution of the radial size of the polymer, and (b) the variation of the polymer conformation with time during the stretching process for N=130 at $\dot{\varepsilon}_H=5\times10^{-2}$. Polymers A, B, C, and D are chosen to represent small deformations at $\varepsilon_H=2$. Their radial sizes are located in the neighborhood of the peak with smaller radial sizes.

(i) R_g of polymer A decreases gradually with time because of compression along the direction perpendicular to the stretching direction.

(ii) R_g of polymer B declines first but grows eventually because of compression in one direction but stretching in the other direction.

(iii) R_g of polymer C is elongated initially but then arrested in a specific stretched state.

(iv) R_g of polymer D is elongated initially but returns to the weakly stretched state via local relaxation.

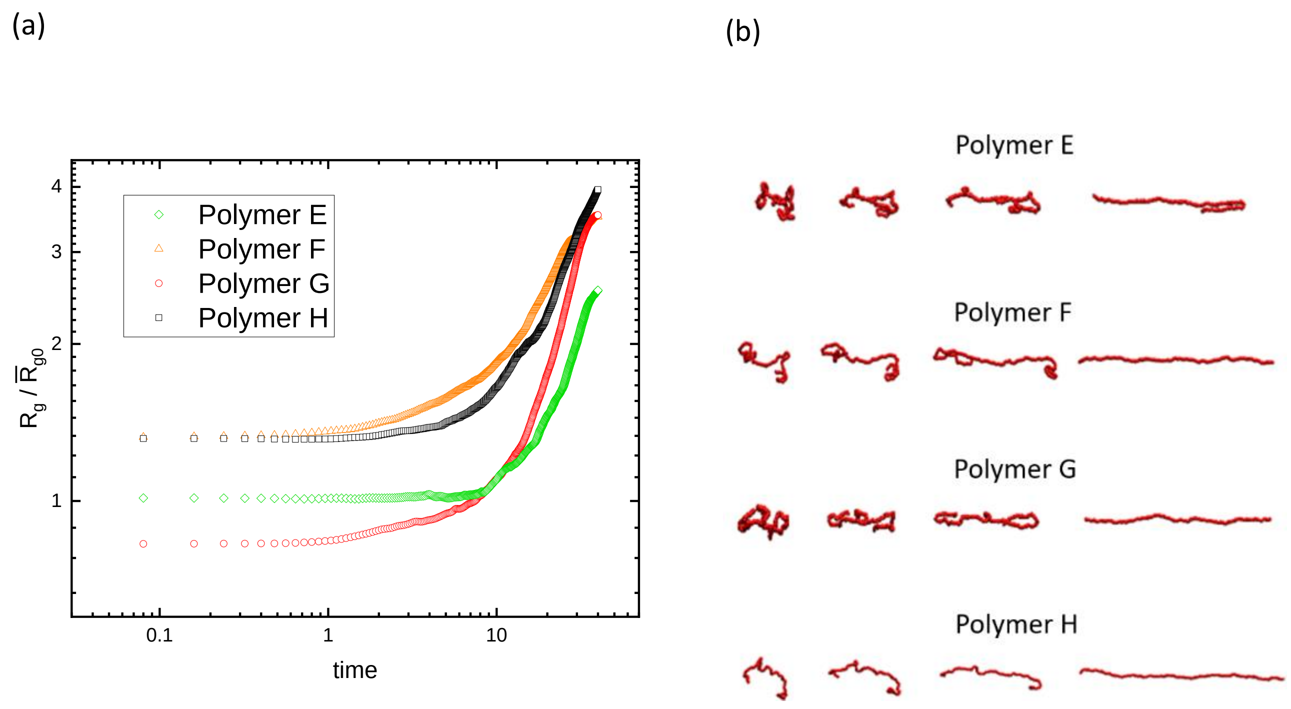

Figure S2. (a) The representative evolution of the radial size of the polymer, and (b) the variation of the polymer conformation with time during the stretching process for N=130 at $\dot{\varepsilon}_H=5\times10^{-2}$. Polymers E, F, G, and H are chosen to represent large deformations at $\varepsilon_H=2$. Their radial sizes are located in the neighborhood of the peak with larger radial sizes. Although those polymers have different initial values of R_g, they are continuously stretched along the stretching direction. While polymer E is still partially stretched, the rests are close to the fully stretched state.
